# Pseudoendocrine sarcoma: a rare new entity with unique radiologic and pathologic/molecular characteristics

**DOI:** 10.1007/s00256-024-04753-w

**Published:** 2024-08-02

**Authors:** Zachary Corey, Julie C. Fanburg-Smith, Cristy N. French, Eric A. Walker, Harry N. Kamerow, Eric L. Cochran, Jessica D. Smith, Donald J. Flemming, Mark D. Murphey

**Affiliations:** 1 UCHealth at University of Colorado Anschutz Medical, Aurora, CO USA; 2 Department of Pathology and Pediatrics, UVA Health University of Virginia Medical Center, Charlottesville, VA 22903 USA; 3 Departments of Orthopedic Surgery, Penn State Health, Hershey, PA 17033 USA; 4 Department of Radiology, Penn State Health, Hershey, PA 17033 USA; 5 Mount Nittany Medical Center, State College, PA 16803 USA; 6 MSII WVSOM, Lewisburg, WV 24901 USA; 7 Radiology Department, University of Kentucky College of Medicine, Lexington, KY 40546 USA; 8 ACR – Institute for Radiologic Pathology (AIRP), Silver Spring, MD 20910 USA; 9 Department of Pathology, Penn State Health, Hershey, PA 17033 USA

**Keywords:** Radiologic feeder vessels, Pathologic vascularity/zellballen pattern, Pseudoendocrine sarcoma, *CTNNB1* point mutation, Diagnosis, Rare new entity

## Abstract

Pseudoendocrine sarcoma is a rare, recently described intermediate grade sarcoma of uncertain phenotype that most commonly affects the paraspinal location in older patients with a distinctive endocrine/paraganglioma-like morphology and unique *CTNNB1* point mutation. While these tumors appear as epithelial or even benign endocrine tumors, these lack markers for such and are highlighted by nuclear expression of beta-catenin. This case is the first among the previously reported only twenty-five cases of this entity, including one original series and a few case reports, to correlate the radiologic imaging with the pathologic features. Furthermore, this case illustrates the oldest-to-date patient with this unique location as a palpable painful chest wall/paraspinal location, with new morphologic observations and, finally, this is only the second case to have this specific *CTNNB1* hotspot point mutation for this rare entity.

## Introduction

Pseudoendocrine sarcoma is a newly described, rare entity, usually located on the paraspinal trunk in older adults. Since the seminal original series of 23 cases reported in January 2022, there have only been two additional single case reports, making this the twenty-sixth case described. Albeit rare, this distinctive pseudoendocrine sarcoma appears to have reproducible clinical and histopathologic features that are identified in the current case and those in the literature. It is most important to separate this intermediate grade sarcoma from benign endocrine mimickers. Unique to the current case are the radiologic imaging correlation of pseudoendocrine sarcoma, newly herein described are the increased vascularity and feeding vessels noticed by our radiologists also, this is the oldest patient with this entity in a “chest wall” paraspinal location, clinical features helpful for surgeons and other clinicians. The distinctive type of *CTNNB1* hotspot mutation, this case is the second case to have this particular unique mutation, and new morphologic observations separate from other beta-catenin nuclear-positive tumors, a fact useful for pathologists. It is important for all interdisciplinary members of the musculoskeletal team to recognize these distinctive features of this intermediate grade sarcoma, for best correlative diagnosis and patient management.

## Case report

An 82-year-old female presented to an outside health center with a painful right chest wall mass. A subsequent chest radiograph and ultrasound-guided core needle biopsy were performed. Pathologic diagnosis was initially unclear, as this new sarcoma entity is rare and newly described and can mimic a benign or metastatic epithelial or endocrine tumor. This patient’s biopsy and imaging studies were sent to our institution for consultation.

**Fig. 1:**
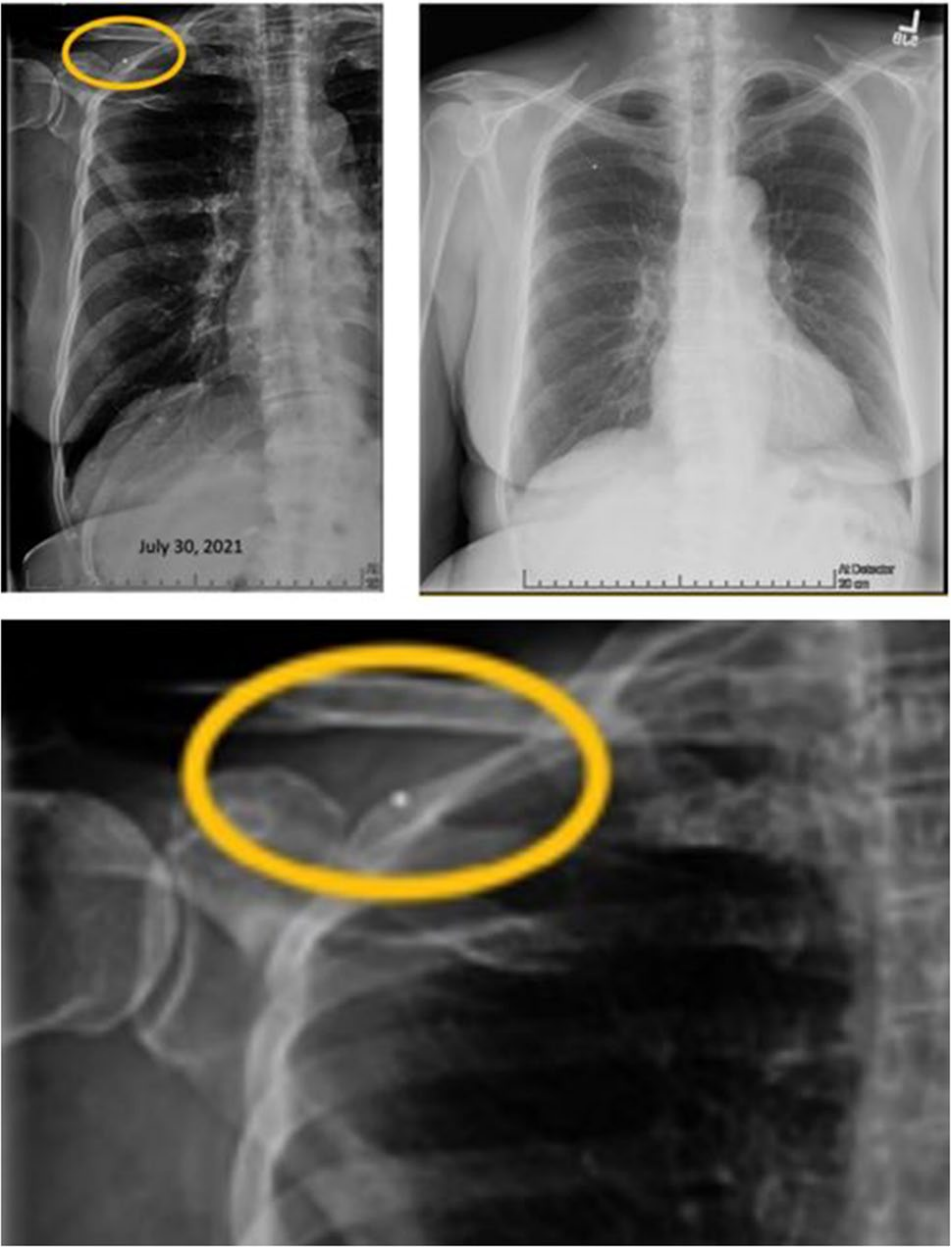
AP chest radiograph and higher coned down view of AP chest radiograph revealed a subtle right upper chest wall soft tissue density (yellow circle) indicated by the metallic marker denoting a palpable mass. Note the absence of rib destruction, rendering this lesion difficult to discern at all

**Fig. 2:**
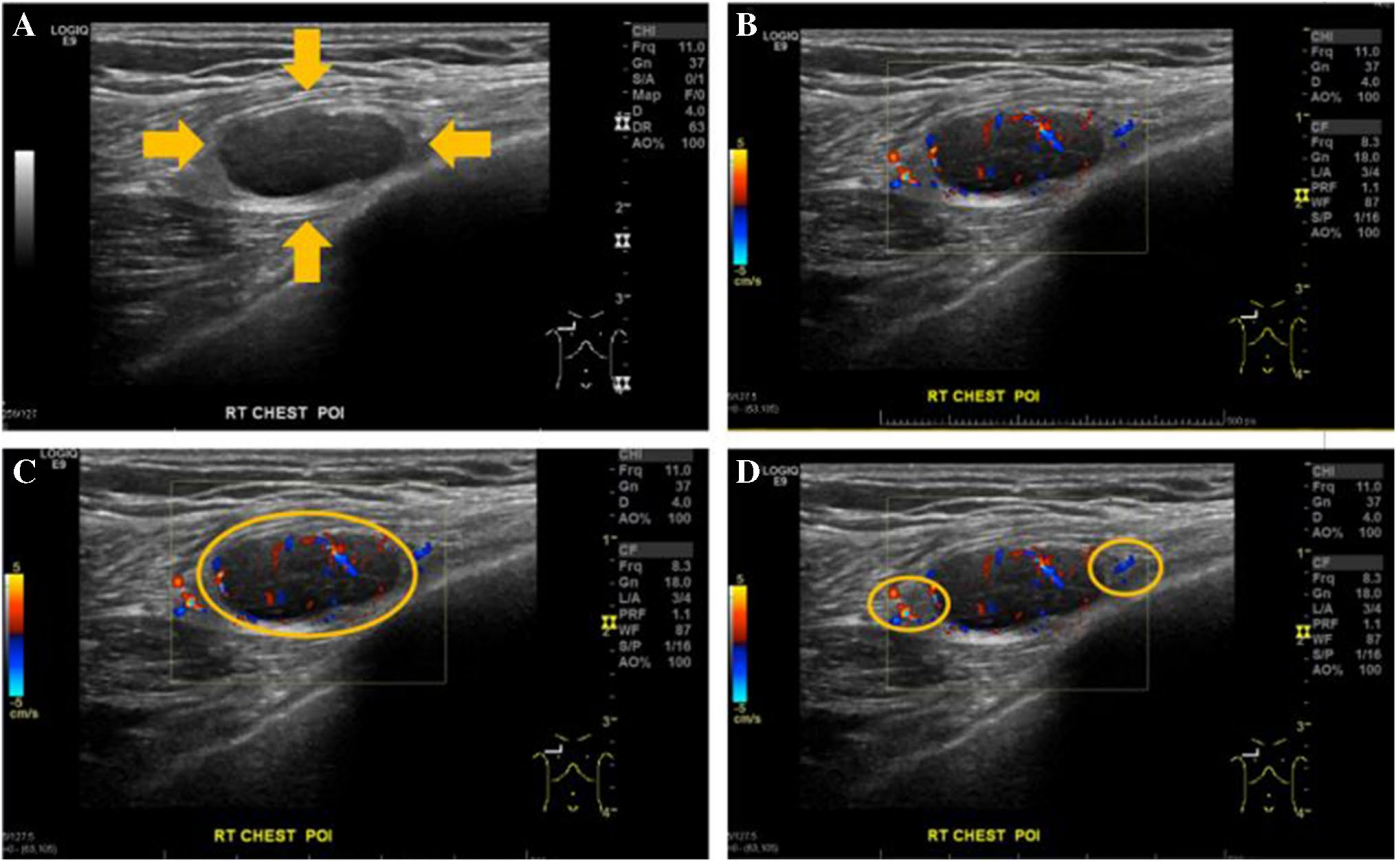
Ultrasound of chest wall mass. **A** Obtained due to pain in the area despite the only vague radiograph findings. Gray-scale ultrasound demonstrates a sharply demarcated, homogeneous, hypoechoic chest wall intramuscular mass without internal calcification (arrows). **B**–**D** Color Doppler imaging (**B**–**C**) demonstrates internal vascularity (large yellow circle) and possible peripheral feeding vascular pedicle (smaller yellow circles in **D**)

AP chest radiograph revealed a subtle right upper chest wall soft tissue density mass without osseous destruction and therefore was difficult to discern (Fig. 1). Following the vague suggestion of a mass and corresponding soft tissue density noted on chest radiograph, an ultrasound-guided core needle biopsy was performed. The preprocedural ultrasound revealed an oval, well-circumscribed, homogeneous hypoechoic intramuscular mass. No internal calcification or cystic components were identified. Internal vascularity was observed within the mass on color Doppler imaging (Fig. 2) with possible feeding vascular pedicles, a subtle clue to diagnosis, in addition to the circumscription and location and presentation, requiring pathology and molecular to confirm the final diagnosis.

**Fig. 3:**
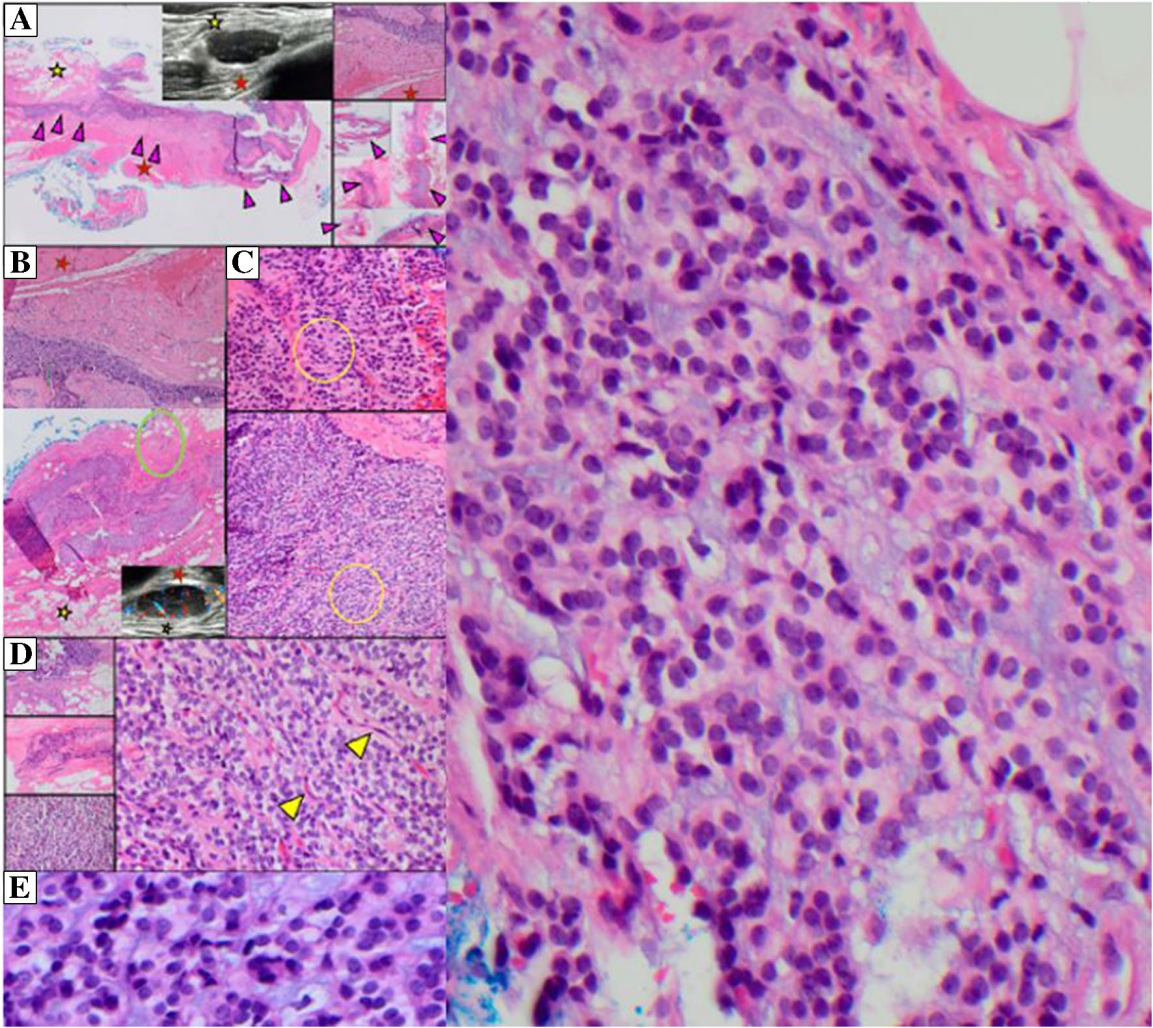
Representative images of pseudoendocrine sarcoma pathology with radiologic correlation. **A** Lobular, infiltrative pattern (purple arrowheads) correlated to ultrasound imaging (yellow and red stars). **B** Feeder vessels (green oval) correlated to ultrasound imaging (yellow and red stars). **C** Nestled growth pattern with uniform epithelial/ovoid cells (yellow circle). **D** Round nuclei with speckled well-dispersed chromatin (right picture) with rare mitoses and no necrosis, the vessels (yellow arrowheads) create the zellballen pattern. Note in (**E** and to the right) a loss of zellballen pattern to trabecular with of oval/round cells with well-dispersed chromatin, with focal cytoplasmic clearing/rare paranuclear vacuole, distinctive cytoplasmic borders, and unique myxoid stroma, confusing this tumor with other entities

The histopathologic features (Fig. 3) of this small 3-cm mass reveal a lobular, well delineated, and circumscribed ovoid-shaped tumor with a pseudocapsule. There was a combination of pseudoendocrine/paraganglioma-like features with peripheral capillary vessels, creating the zellballen pattern, admixed with a trabecular (also pseudoendocrine/paraganglioma-like) appearance composed of round to ovoid cells with well-dispersed chromatin and distinctive cytoplasmic borders. The cells demonstrated focal clear cell to cytoplasmic vacuole-appearing features within myxoid stroma that lacked the zellballen pattern, without extracellular psammomatous calcifications, hyaline globules, metaplastic bone, or necrosis. Mitoses were not significantly increased, and there were no atypical mitoses.

**Fig. 4:**
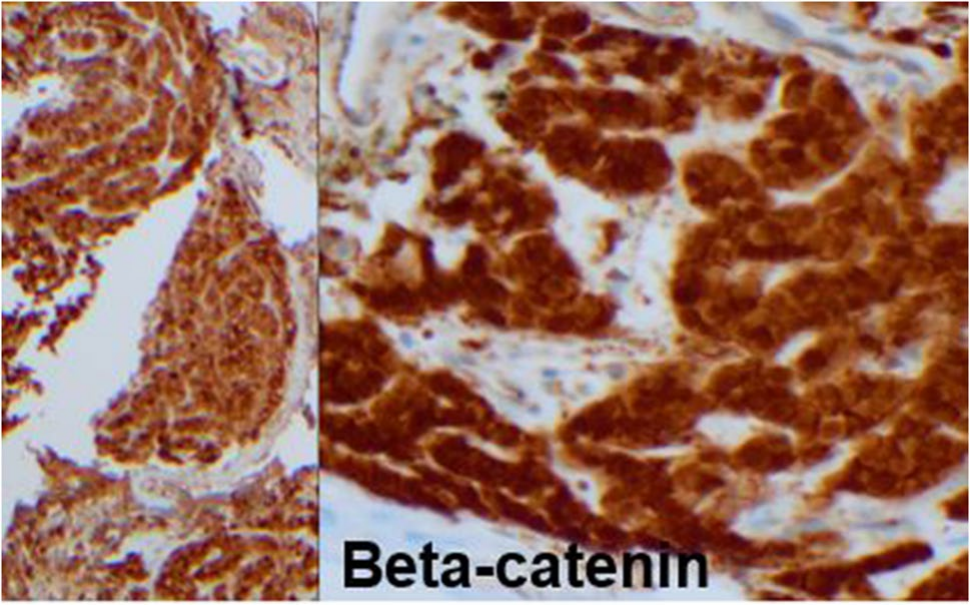
Beta-catenin immunostain of intermediate grade sarcoma pseudoendocrine sarcoma. Positive nuclear beta-catenin corresponds to the specific *CTNNB1* (c98C > G, p.Ser33Cys in exon 2) hotspot point mutation, only the second case of pseudoendocrine sarcoma to exhibit this specific hotspot mutation and distinctive from those *CTNNB1* hotspot point mutations in other entities such as desmoid fibromatosis

Immunostaining was initially ordered by the original pathologist and was positive for nonspecific CD10 and CD99, with mild to focally moderate MIB-1 (indicating intermediate grade sarcoma) and negative for keratin, EMA, MUC4, synaptophysin, chromogranin, and S100 protein (no sustentacular cells), CD34, SMA, desmin, CD68, CD138, CD117, DOG1, GATA-3, CAIX, MITF, Melan-A, and TFE3, excluding endocrine tumors such as benign paraganglioma or malignant adrenal cortical carcinoma and epithelial and melanocytic tumors. A comprehensive sarcoma NGS fusion panel that included *BCOR*, *FUS (SEF)*, *GLI1*, *NOTCH1/2*, *PHF1*, and *STAT6* was negative, failing to reveal a specific diagnosis. The case was sent in consultation and during consultative workup, a nuclear beta-catenin was ordered to exclude this new entity and was positive (Fig. 4, beta-catenin) and a desmoid hotspot panel revealed a unique somatic point mutation for *CTNNB1* (c98C > G, p.Ser33Cys in exon 2, hotspot point mutation *S33C*), supporting the diagnosis of pseudoendocrine sarcoma, the second only of these tumors to exhibit this exact hotspot point mutation.

Based on the clinicoradiologic, morphologic, and phenotypic features with *CTNNB1* hotspot mutation, a final diagnosis of pseudoendocrine sarcoma was rendered. The patient was treated by complete excision of the mass. Follow-up of more than 4 years demonstrated no evidence for local recurrence or metastasis.

## Discussion

Pseudoendocrine sarcoma is a distinctive, unique low to intermediate grade tumor, most commonly affecting the paraspinal soft tissue in older adults, with recurrent and metastatic potential [1–3]. Hypervascularity/possible vascular pedicle on radiologic imaging correlates with a vascular driven-zellballen pattern microscopically [4]. The round cellular features with well-dispersed chromatin resemble endocrine/paraganglioma-like and sclerosing epithelioid fibrosarcoma-like entities. The myxoid stroma also raises consideration of a pericytoid, myoepithelioid, or even extraskeletal myxoid chondrosarcoma, myoepithelioma, sclerosing epithelioid fibrosarcoma, or other tumor with chondromyxoid features in the differential diagnosis; morphology combined with immunophenotypic markers do not confirm these entities. When chromogranin and synaptophysin are negative with these paraganglioma-like features, a positive nuclear B-catenin and a point mutation of *CTNNB1* can separate this tumor from radiologic and morphologic cellular and stromal mimickers. Pseudoendocrine sarcoma has herein CD10 positivity and reported LEF1 reactivity (involved in *Wnt/CTNNB1* pathway) and distinctive lack of other markers including for epithelial, neuroendocrine, adnexal, or perineurial/meningothelial differentiation, despite overlapping ultrastructural features noted with the latter.

Our reported case is in the oldest patient to date, at 82 years, diagnosed with pseudoendocrine sarcoma, while the average patient age from reported literature is 60, with a range up to 72 years. In addition, the current patient has one of the smallest tumor sizes of 3 cm (with the reported average being 6.7 cm) and a reported upper chest wall/paraspinal location, similar to the other reported pseudoendocrine sarcomas in the paraspinal region. Although our tumor has not recurred or metastasized, there is a reported high rate of local recurrence (43%) and lymph node and pulmonary metastases (21%) for this entity [1–5], classifying this entity as an intermediate grade sarcoma.

None of the limited series of 23 cases or single case reports of pseudoendocrine sarcoma correlate the radiologic findings in detail [1, 3, 5]. The radiologic findings on Doppler ultrasonography in our case demonstrated increased vascularity and feeder pedicle with internal vascularity that nicely corresponds to the feeder vessels and zellballen pattern delimited by the surrounding vascularity by morphology. These flow features bring other high flow tumors into the radiologic imaging differential diagnosis, including soft tissue angiofibroma, solitary fibrous tumor, synovial sarcoma, and alveolar soft part sarcoma [4], excluded by morphology, phenotype, and genetic/molecular findings.

Nuclear beta-catenin and point mutation of *CTNNB1* can also be identified in desmoid fibromatosis and other Gardner syndrome *APC/Wnt*-pathway tumors. Several other neoplasms also harbor *CTNNB1* point mutations including endometrial, hepatobiliary, and colorectal carcinomas and melanoma. The exact point mutations of *CTNNB1* of pseudoendocrine sarcoma appear to be distinctive, mainly in exon 2 (in our case *S33C*), and differ from desmoid fibromatosis, which is mainly in exon 3 and most commonly *T41A* (Table 1) [6–9].

**Table 1:** Comparison of *CTNNB1* mutation sites in pseudoendocrine sarcoma and desmoid fibromatosis, with no overlap between the two entities. In this case, S33C* hotspot mutation is present, as only the second case of pseudoendocrine sarcoma to harbor this exact hotspot mutation

Tumor type	*CTNNB1* mutation sites
Pseudoendocrine sarcoma^1−3^	S33C* *S33F*
	*S37C*
	*S37F*
	*D32H*
	*S37A* *S33P*
Desmoid fibromatosis^5−8^	*T41A*
	*T41I*
	*S45F*
	*S45P*
	*S45C*

In summary, this is the twenty-sixth case of newly described pseudoendocrine sarcoma in the chest wall/soft tissue of an elderly woman, diagnosed by having a benign endocrine-like appearance with a complete workup that excluded other entities. The distinctive clinical features, morphologic and phenotypic features with negative markers-favored an intermediate grade sarcoma that was originally negative for a complete sequencing workup. However, the final recognition of this rare novel entity was by a combination of observations by surgeons, radiologists, and pathologists to recognize this rare entity and obtain the final positive nuclear beta-catenin and distinctive *CTNNB1* point mutation. Important are the unique features of the current case and the radiologic vascularity that corresponds to microscopic pathologic (Zellballen) vascular features, which resulted in proper diagnosis and management of this potentially metastasizing intermediate grade sarcoma.

## Data Availability

All data available on this case is reported within this publication.
